# Whole-mount diffracted X-ray blinking analysis of intracellular amyloid-β-associated nanoscale dynamics in *Caenorhabditis elegans*

**DOI:** 10.1093/biomethods/bpag044

**Published:** 2026-08-13

**Authors:** Kotaro Ozaki, Yoichi Shinkai, Yuji C Sasaki, Masahiro Kuramochi

**Affiliations:** Graduate School of Science and Engineering, Ibaraki University, Hitachi 316-8511, Japan; Cellular and Molecular Biotechnology Research Institute, National Institute of Advanced Industrial Science and Technology (AIST), Tsukuba 305-8566, Japan; Graduate School of Frontier Sciences, The University of Tokyo, Kashiwa 277-8561, Japan; Graduate School of Science and Engineering, Ibaraki University, Hitachi 316-8511, Japan; Graduate School of Frontier Sciences, The University of Tokyo, Kashiwa 277-8561, Japan

**Keywords:** diffracted X-ray blinking, amyloid-β, *Caenorhabditis elegans*, neurodegeneration, molecular dynamics

## Abstract

Nanoscale protein dynamics are closely associated with molecular function and pathological structure formation, yet their measurement within animal tissues remains technically challenging. In this study, we developed a diffracted X-ray blinking (DXB) method to evaluate amyloid-β (Aβ)-associated nanoscale fluctuations in fixed and permeabilized whole-mount *Caenorhabditis elegans*. Intracellular Aβ expressed in neurons (nAβ) or body-wall muscle cells (mAβ) was labelled with gold nanoparticles using an antibody-mediated strategy, without dissection or sectioning. The labelled worms produced detectable Au(111) diffraction signals, and the resulting time-resolved diffraction intensity fluctuations were analysed by autocorrelation function (ACF) analysis. A comparison of the ACF decay constants obtained from nAβ and mAβ worms revealed that compared with nAβ, mAβ contained a greater fraction of fast relaxation components. Furthermore, in-vitro DXB measurements of Aβ revealed local fast relaxation components that changed over time in association with changes in Aβ assemblies, partly resembling the Aβ-associated motional features observed in worms. These findings suggest that the detected relaxation components may reflect structurally diverse Aβ states arising during aggregation. These results demonstrate the feasibility of applying DXB to detect Aβ-associated nanoscale fluctuation signatures in fixed whole-mount *C. elegans* and provide a methodological framework for analysing protein-associated dynamic states in animal tissue samples.

## Introduction

Protein dynamics are essential for understanding structure-based molecular function. However, observing molecular motion at the atomic or nanometre spatial scale remains challenging, particularly in cellular environments or whole-mount organisms. Time-resolved X-ray diffraction provides a unique approach for detecting biomolecular motion with extremely high spatial sensitivity. We previously developed diffracted X-ray blinking (DXB), a time-resolved X-ray diffraction method based on monochromatic X-rays [1]. In DXB, angular fluctuations of nanocrystal-labelled molecules are detected as time-dependent changes in diffraction intensity. Diffraction spots appear or disappear as the nanocrystal enters or leaves the Bragg diffraction condition, and this blinking behaviour reflects tilting and twisting motions of the labelled molecule. These motions can be quantified by autocorrelation function (ACF) analysis of the time-dependent diffraction intensity.

DXB has been applied to a wide range of biological and material systems, including biomacromolecules such as TRPV channels, acetylcholine receptors, ice-binding proteins and viral spike proteins, as well as silver halide crystals, photoresponsive materials and rubber materials [1–7]. One of the major advantages of DXB is that molecular motion can be detected over relatively long-time scales using a low X-ray dose. Furthermore, because DXB can be performed not only at synchrotron radiation facilities but also using laboratory X-ray sources, it has the potential to serve as a versatile platform for molecular dynamics measurements. Previous studies have shown that DXB can detect protein motion in-vivo or in biologically relevant environments, including molecular motion measurements in the nematode *Caenorhabditis elegans* [3]. However, these measurements have focused mainly on targeted molecules exposed to the extracellular environment or membrane-associated proteins that are accessible from outside the cell. In contrast, methods for targeting intracellular molecules and measuring their nanoscale dynamics remain insufficiently established.

Intracellular proteins exist in complex environments characterized by molecular crowding and local heterogeneity [8–10]. Their molecular motion can be strongly influenced by their location, binding partners, aggregation state, and surrounding physicochemical environment [11, 12]. Therefore, extending DXB to intracellular targets represents an important step towards evaluating protein dynamics under more physiologically relevant conditions. Amyloid-β (Aβ) is a suitable target for this purpose. The molecular state of Aβ changes during aggregation and assembly, and these changes are closely associated with cellular dysfunction and neurodegenerative disease [13–15]. We previously performed X-ray dynamics measurements of in vitro Aβ and reported a relationship between the molecular motion of Aβ and its aggregation process [16]. In addition, *Caenorhabditis elegans* is a genetically tractable animal model in which Aβ can be expressed in specific tissues, allowing for intracellular Aβ dynamics and whole-animal phenotypes to be evaluated in parallel [17–19].

**Figure 1:**
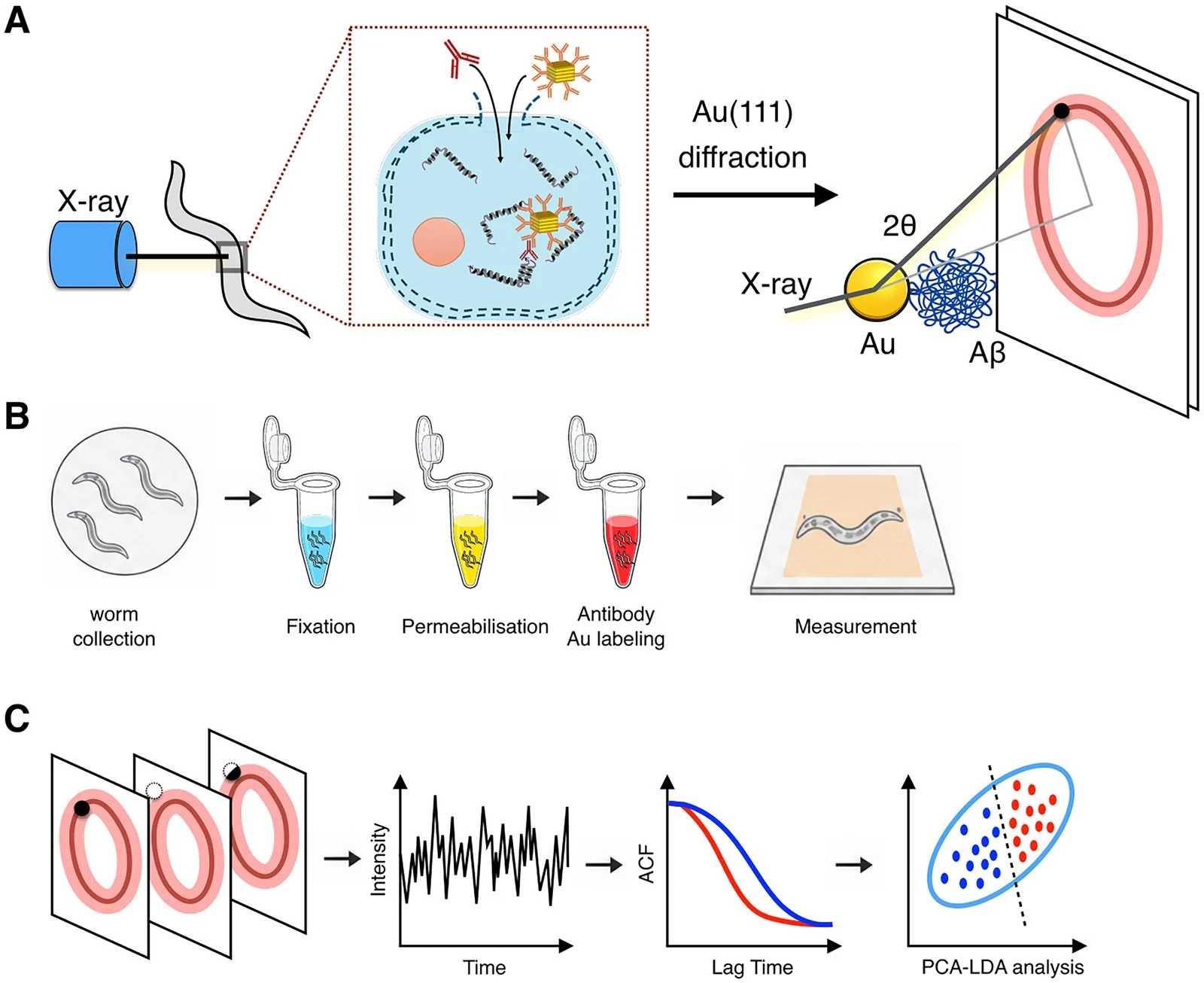
Conceptual overview of DXB measurement and analysis of intracellular Aβ dynamics in *C. elegans*. (A) Schematic illustration of diffracted X-ray blinking (DXB) measurements of intracellular Aβ in *C. elegans*. Aβ molecules expressed inside worm cells are labelled with gold nanocrystal-conjugated antibodies. Upon X-ray irradiation, Au(111) diffraction from the gold nanocrystal-labelled Aβ complex is recorded on the detector. Angular fluctuations of the labelled Aβ complex change the diffraction condition in the χ and/or 2θ directions, resulting in diffraction intensity fluctuations or “blinking”. (B) Experimental workflow for preparing gold-labelled worms. Worms are collected, fixed, permeabilized, labelled with antibody–gold nanocrystal complexes, and mounted for DXB measurement. (C) Analysis workflow of the DXB signals. Time-dependent fluctuations in diffraction intensity are extracted from sequential diffraction images, converted into autocorrelation functions (ACFs), and further analysed by PCA–LDA to evaluate differences in the relaxation behaviour of intracellular Aβ.

In this study, we developed a DXB-based method for measuring Aβ-associated nanoscale dynamics in fixed and permeabilized whole-mount transgenic *C. elegans* samples (Fig. 1A). Inspired by conventional fluorescence immunostaining, Aβ molecules expressed in neurons or body-wall muscle cells were labelled with gold nanoparticles through antibody-mediated labelling after fixation and permeabilization, without dissection or sectioning of the worms (Fig. 1B). Time-resolved Au(111) diffraction signals derived from the gold nanoparticles were then analysed by ACF analysis to detect local rotational and fluctuational motions near Aβ. In addition to conventional ACF fitting, principal component analysis and linear discriminant analysis were applied to compare the dynamic features of the neuronal and muscle Aβ samples (Fig. 1C). We also performed in vitro DXB measurements of Aβ as reference data for interpretation and evaluated physiological phenotypes, including locomotion, egg laying, and NaCl-associated learning. This study demonstrates the feasibility of using DXB to detect Aβ-associated nanoscale dynamics in fixed whole-mount *C. elegans* samples and provides a methodological basis for analysing intracellular protein-associated dynamics in animal model systems.

## Materials and methods

### Molecular biology, transgenic worms, and culture conditions

To express human amyloid-β 1–42 peptide (Aβ1–42) in body-wall muscle cells or the nervous system of *C. elegans*, we used the *myo-3* or *H20* promoter, respectively. The expression constructs were generated using pPD95.79, which was kindly provided by Dr. Andrew Fire, as the backbone. A chemically synthesized Aβ coding sequence was inserted into the EcoRI site of a promoter-containing fragment. The vector was treated with calf intestinal phosphatase to prevent self-ligation and subsequently ligated with the Aβ insert to generate the final plasmid construct. The orientation and sequence of the inserted Aβ fragment were confirmed by DNA sequencing.

Transgenic *C. elegans* strains expressing Aβ were generated by standard microinjection methods [20]. The strains used in this study were as follows:

CMS50 Ex[*H20p::Xpa H62Q* (100 ng/µL); *lin-44p::mCherry* (30 ng/µL)], CMS80 Ex[*myo-3p::Aβ* (50 ng/µL); *lin-44p::mCherry* (30 ng/µL)], CMS95 Ex[*myo-3p::wrmScarlet* (50 ng/µL); *lin-44p::mCherry* (30 ng/µL)], and CMS99 Ex[*H20p::Aβ* (50 ng/µL); *lin-44p::mCherry* (30 ng/µL); *baf-1p::sat2* (20 ng/µL)].

Worms were maintained on nematode growth medium (NGM) agar plates with *Escherichia coli* OP50 at 24°C.

### Aβ toxicity assays

**Figure 2:**
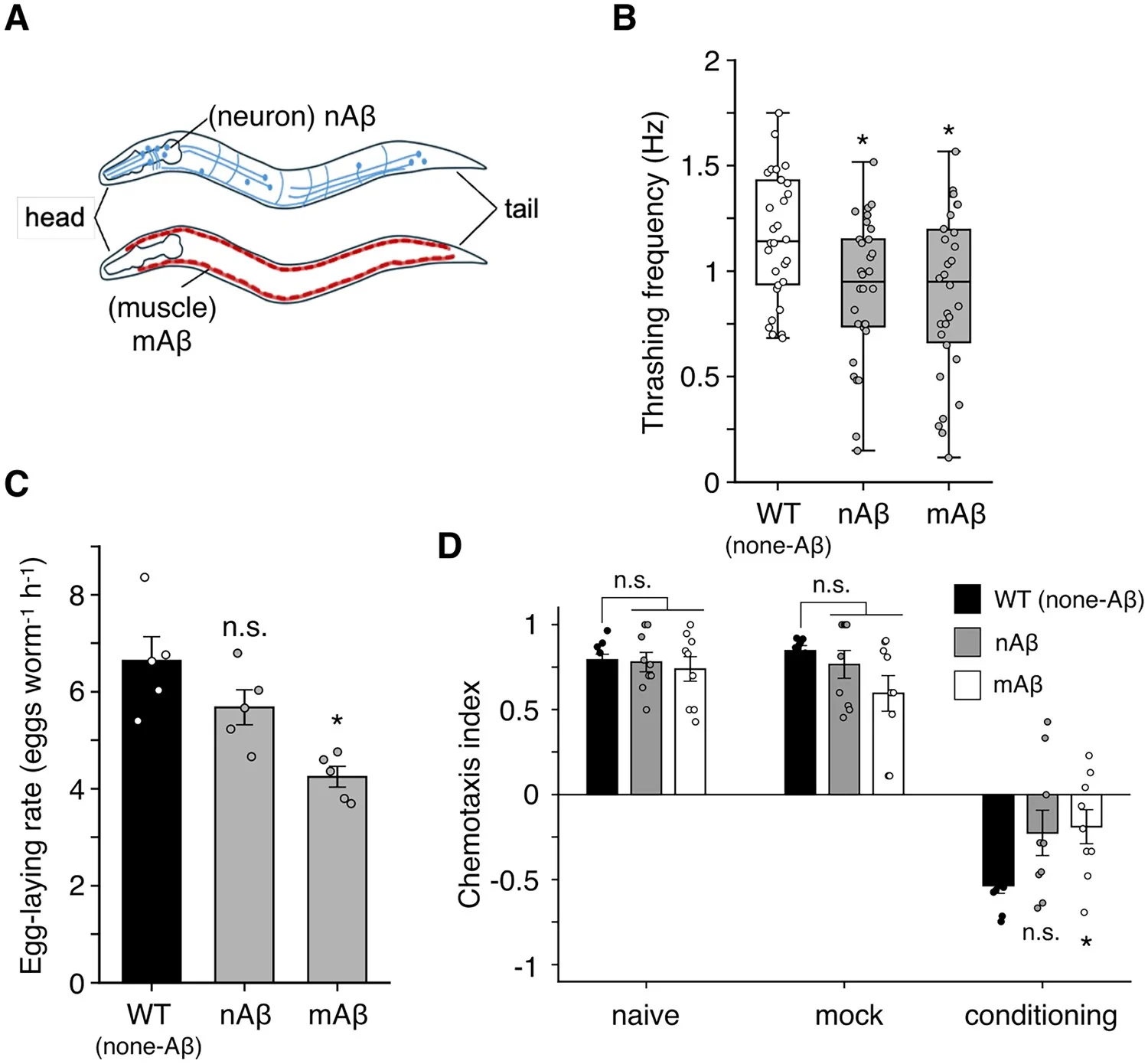
Physiological effects of neuronal and muscle Aβ expression in *C. elegans*. (A) schematic illustration of *C. elegans* strains expressing Aβ in neurons (nAβ) or body-wall muscle cells (mAβ). (B) Thrashing frequency of wild-type (WT), neuronal Aβ-expressing and muscle Aβ-expressing worms. Wilcoxon rank-sum test. *n* = 30. (C) Number of eggs laid by WT, neuronal Aβ-expressing and muscle Aβ-expressing worms. Five independent assays were performed, each using 10 worms. (D) NaCl associative learning assay after 45 min of conditioning. Five independent assays were performed, each using 50–300 worms. The data are presented as the means ± SEM. Statistical significance was assessed using Student’s *t*-test. *P < .05; n.s., not significant.

To evaluate the effects of Aβ expression in neurons (nAβ) and body-wall muscle cells (mAβ), transgenic worms expressing Aβ in either the nervous system or body-wall muscle cells were used (Fig. 2A). Thrashing, egg laying, and NaCl learning assays were performed. For all the assays, the worms were age-synchronized by allowing for 10 adult worms per plate to lay eggs for 4 h, after which the Day 4 adults after egg laying were used for analysis.

For the thrashing assay, 10 µL of M9 buffer was placed on a glass slide, and a single worm was transferred into the droplet. After 30 s of acclimation, the number of thrashes was counted for 1 min. One thrash was defined as one complete left–right bending cycle of the body. Thirty worms were analyzed for each group. Statistical analysis was performed using the Wilcoxon rank-sum test.

For the egg laying assay, 10 adult worms were transferred to a fresh NGM plate seeded with OP50 and allowed to lay eggs for 3 h. The adult worms were then removed, and the number of eggs laid on the plate was counted under a stereomicroscope. The results are expressed as the number of eggs per worm per hour and were compared among groups. Five independent experiments were performed for each group, and statistical analysis was carried out using Student’s *t*-test.

For the NaCl learning assay, age-synchronized adult worms were used, and learning ability was evaluated by comparing N2, nAβ, and mAβ worms under naive, mock, and conditioning conditions. Assay plates were prepared in 6-cm dishes using agar supplemented with 5 mM KHPO_4_, 1 mM MgSO_4_, 1 mM CaCl_2_, and 2% Difco Bacto agar. NaCl plugs were prepared using the same medium supplemented with 100 mM NaCl. The wash buffer consisted of 5 mM KHPO_4_, 1 mM MgSO_4_, 1 mM CaCl_2_, and 0.005% gelatine, and the conditioning solution consisted of the same buffer supplemented with 50 mM NaCl. On the day before the assay, a NaCl plug was placed at the designated position on the assay plate and left for 16–24 h to establish a NaCl concentration gradient. On the day of the assay, the worms were collected and washed with wash buffer and then divided into three groups: naive, mock, and conditioned groups. The naive group was subjected to the assay immediately after collection. The mock group was incubated in wash buffer, whereas the conditioning group was incubated in conditioning solution, each for 45 min at 24°C. After treatment, the worms were washed with wash buffer and used for the assay. At the start of the assay, the NaCl plug was removed, and 1 µL of 50 mM NaN_3_ was applied to both the side where the NaCl plug had been placed and the opposite side. Worms were placed at the centre of the plate and left for 30 min at 24°C. The numbers of worms in the region on the NaCl plug side (A) and the opposite side (B) were then counted, and the chemotaxis index was calculated as (A-B)/(A+B). The same equation was also used for the chemotaxis assay. Five independent experiments were performed for each group, with approximately 50–300 worms used in each trial. Statistical analysis was performed using Student’s *t*-test.

### Gold nanoparticle labelling of amyloid-β and sample preparation for X-ray measurements

*C. elegans* expressing Aβ were collected from agar plates using M9 buffer and transferred to 1.5-mL tubes. The worms were subsequently centrifuged at 3000× *g* for 3 min, after which the supernatant was removed. The worm pellet was then washed three times with M9 buffer to remove residual *E. coli*. Finally, the worms were resuspended in a small volume of M9 buffer. The worms were fixed, permeabilized, immunolabelled, and labelled with gold nanoparticles in tubes without dissection or sectioning, thereby preserving the overall body structure. For immunolabelling of Aβ, worms were fixed with 4% paraformaldehyde (PFA) in Phosphate Buffered Saline (PBS) for 30–60 min at room temperature. After fixation, the worms were washed three times with PBS. To allow for the antibody and gold nanoparticle-labelled secondary antibody to penetrate into the worms, the fixed worms were treated with SDS/β-mercaptoethanol. The permeabilization solution contained 1% SDS, 1% β-mercaptoethanol, 0.1 M Tris-HCl (pH 7.5), 0.9% NaCl and 1 mM EDTA. Worms were incubated in this solution at 37°C for 30–45 min with gentle mixing. The treatment time was adjusted to increase cuticle permeability while avoiding excessive damage to worm morphology. After permeabilization, the worms were washed four to five times with PBST, which consisted of PBS supplemented with 0.1% Triton X-100. To reduce nonspecific binding, the worms were blocked in PBS containing 1% BSA and 0.1% Triton X-100 for 30–60 min at room temperature. The anti-Aβ42/43 primary antibody, BC05, was then diluted 1:200 in blocking buffer, added to the worm pellet, and incubated overnight at 4°C with gentle rotation. After primary antibody incubation, the worms were washed four to five times with PBST for 5–10 min per wash.

For some samples, fluorescence immunostaining was performed to confirm Aβ labelling. Worms labelled with the BC05 primary antibody were incubated with a fluorescently labelled anti-mouse IgG secondary antibody, washed with PBST, and observed by fluorescence microscopy. These fluorescence observations were used as a preliminary validation step before DXB measurements were performed using gold nanoparticle labelling.

For DXB measurements, the primary antibody bound to Aβ was labelled with a gold nanoparticle-conjugated anti-mouse IgG secondary antibody. The secondary antibody was diluted 1:20 in PBST and incubated with the worms for 1–2 h at room temperature in the dark with gentle rotation. Because the Au(111) diffraction signal obtained using the gold nanoparticle-conjugated secondary antibody alone was weak, an additional incubation with approximately 60-nm gold nanoparticles was performed to enhance the X-ray scattering contrast. This additional labelling step was introduced while considering both the penetration of particles into the permeabilized worms and the need to obtain sufficient Au(111) diffraction intensity for DXB analysis. Although this procedure may increase the nonspecific retention of gold nanoparticles, non-Aβ control worms were analysed in parallel to evaluate the background signal.

After gold nanoparticle labelling, the worms were washed four to five times with PBST, followed, when necessary, by additional washes with PBS. For X-ray measurements under dry or low-salt conditions, the final wash was performed once or twice with distilled water to suppress salt crystal formation. The labelled worms were then placed on a polyimide film and mounted on a sample holder. The worm concentration was adjusted to avoid excessive overlap between individuals. The mounted samples were subsequently subjected to X-ray measurements.

### Preparation of amyloid-β samples for in vitro DXB measurements

Aβ was dissolved in 0.1% NH_4_OH/1 mM DMSO and prepared at a final concentration of 0.1 mg/mL. The Aβ solution was incubated at 37°C for 0, 90, or 180 min or for 24 h. Immobilization of Aβ molecules on the substrate film and labelling with gold nanoparticles were performed on the basis of a previously reported method [16]. To immobilize Aβ on a polyimide film by amine coupling, the film was first treated with KOH. A total of 100 μL of NHS/WSC solution (Dojindo), prepared in minimum essential medium (MEM) buffer, was then added to the KOH-treated film and incubated for 10 min at room temperature. The Aβ solution was diluted to 70 μg/mL in activation buffer, and 100 μL of this solution was applied to the film for 30 min at room temperature. Blocking solution was then added, and the samples were incubated for 30 min at room temperature, followed by washing with PBS. For the 0 min condition, Aβ was immobilized on the substrate film, and the relevant reagents were added immediately before measurement. For the 90- and 180-min conditions, Aβ was immobilized on the substrate film and then incubated at 37°C for 90 or 180 min before measurement. After 24 h, the Aβ solution was first incubated at 37°C for 24 h and then immobilized on the substrate film.

For gold nanoparticle labelling of Aβ, 100 μL of N-succinimidyl 3-(2-pyridyldithio)propionate (SPDP) crosslinker dissolved in DMSO was added to the Aβ-immobilised film and incubated for 60 min at room temperature. Later, 10 mM EDTA and 0.3 mM dithiothreitol in PBS were added to the film and incubated for 30 min at room temperature to introduce thiol groups into SPDP. The 60-nm gold nanoparticles (Cytodiagnostics Inc.) were concentrated by centrifugation at 3000 × *g* for 10 min at 4°C, after which the supernatant was removed. The concentrated gold nanoparticle solution was then applied to the film and incubated for 120 min at room temperature to couple the gold nanoparticles to SPDP via disulfide bonds. The film was then washed with PBS.

### Diffracted X-ray blinking

Time-resolved X-ray diffraction images were acquired either at KEK PF-AR NW12 or using a laboratory X-ray source (MicroMax-007 HF). The diffraction images were recorded with a PILATUS 2M detector at KEK PF-AR NW12 and with a PILATUS 100K-A detector for laboratory measurements (Dectris, Switzerland). Measurements were performed at room temperature. A total of 2010 consecutive X-ray diffraction images were recorded with an exposure time of 50 ms per frame. The incident X-ray beam size used for the measurements ranged from approximately 150 to 800 μm. Time-dependent changes in the Au(111) diffraction intensity were extracted from the acquired image series using ImageJ. A time-dependent linear drift, considered to be instrument-related, was observed in the Au(111) diffraction intensity. Individual diffraction spots typically extended over approximately three to four detector pixels. Therefore, 10 × 10-pixel spatial binning was first applied to improve the signal-to-noise ratio while retaining local diffraction information. The time-dependent diffraction intensity was then extracted from each spatial bin, and the gradual intensity drift was corrected using a linear function before analysis with the following ACF:


$$\mathrm{ACF}(\tau )=\frac{\left\langle I(t)I(t+\tau )\right\rangle }{\langle I(t{)\rangle }^{2}}$$

where I(t) is the diffraction intensity, ⟨⟩ denotes the time average, and τ is the delay time. The obtained ACF curves were fitted with the following exponential function:


ACF(τ)=Aexp⁡(-Γτ)+y

where A is the amplitude, y is the offset, and Γ is the decay constant. Decay constants were accepted when they met the following criteria: (i) y>0, A>0, and Γ>0; and (ii) the fit-residual value (‘chisq’) was at or below the 70th-percentile threshold. These calculations were performed for all 10 × 10-pixel spatial bins in the Au(111) diffraction region.

Each DXB dataset was obtained from a single X-ray measurement of a selected region of one worm. Several positions within each worm were initially scanned, and regions showing clear Au(111) diffraction signals were selected for further analysis. The X-ray beam was directed towards either the neuronal or body-wall muscle region, and the resulting Au(111) diffraction signals were analysed for each 10 × 10-pixel spatial bin. In the DXB analysis, *n* denotes the number of spatial bins for which the ACF curves were successfully fitted, rather than the number of animals or independent biological replicates.

The distribution of decay constants derived from Au(111) diffraction was visualized as histograms and box plots and used to evaluate the dynamic behaviour of protein molecules. Because the distributions of the decay constants did not follow a Gaussian distribution, statistical comparisons were performed using the nonparametric Wilcoxon rank-sum test.

### Principal component analysis and linear discriminant analysis

To evaluate whether the dynamic properties of Aβ differ between muscle and neuronal expression systems, we applied the previously proposed PCA–LDA approach [21]. Principal component analysis (PCA) was performed on the entire ACF dataset to reduce the dimensionality of the ACF profiles. The first 3 principal components were extracted and used as input variables for linear discriminant analysis (LDA), a supervised machine learning method, to compare and classify muscle Aβ and neuronal Aβ samples. For the machine learning analysis, the spatial-bin-level ACF data were divided into two non-overlapping subsets, with one subset used for training and the other for testing. An LDA model was constructed using the PC scores of the training dataset and was then applied to the PC scores of the test dataset. All analyses were performed in a Jupyter Notebook environment using custom-written Python scripts.

## Results

### Physiological effects of Aβ expression in *C. elegans*

We first evaluated the physiological functions of nAβ and mAβ worms using thrashing behaviour, egg laying, NaCl chemotaxis, and NaCl learning as readouts. To assess locomotor function, thrashing behaviour in liquid was measured. The median thrashing frequencies were 1.14 Hz in WT worms, 0.95 Hz in nAβ worms, and 0.95 Hz in mAβ worms (Fig. 2B). Compared with WT worms, both nAβ and mAβ worms presented significantly reduced thrashing frequencies.

Next, to evaluate the effects on reproductive function, egg laying was quantified. The total number of eggs laid was 6.6 ± 0.5 for WT worms, 5.7 ± 0.4 for nAβ worms, and 4.3 ± 0.2 for mAβ worms (Fig. 2C). Compared with that in WT worms, the number of eggs laid was significantly lower in mAβ worms, whereas no significant reduction in egg laying was observed in nAβ worms.

We then examined NaCl chemotaxis and NaCl learning to assess whether Aβ expression affects basal sensory behaviour and experience-dependent behavioural plasticity. Under the naive condition, the chemotaxis indices were 0.79 ± 0.03 for WT worms, 0.78 ± 0.03 for nAβ worms, and 0.74 ± 0.05 for mAβ worms. Under the mock condition, the chemotaxis indices were 0.85 ± 0.06 for WT worms, 0.77 ± 0.08 for nAβ worms, and 0.60 ± 0.13 for mAβ worms (Fig. 2D). No significant differences were observed among WT, nAβ, and mAβ worms under either condition, indicating that Aβ expression did not markedly affect basal chemotaxis in response to NaCl. In contrast, under NaCl learning conditions, in which worms experienced starvation in the presence of NaCl, the chemotaxis indices were −0.53 ± 0.07 for WT worms, −0.23 ± 0.10 for nAβ worms, and -0.19 ± 0.10 for mAβ worms (Fig. 2D). WT worms exhibited learned avoidance of NaCl, whereas this response was significantly attenuated in mAβ worms. A similar trend was observed in nAβ worms compared with WT worms, although the difference did not reach statistical significance.

These results confirm that both nAβ and mAβ worms exhibited physiological abnormalities associated with Aβ toxicity. We therefore considered these worms to be appropriate in-vivo samples for the subsequent evaluation of Aβ-associated nanoscale dynamics by DXB.

### Establishment of gold nanoparticle labelling of intracellular Aβ in *C. elegans*

**Figure 3:**
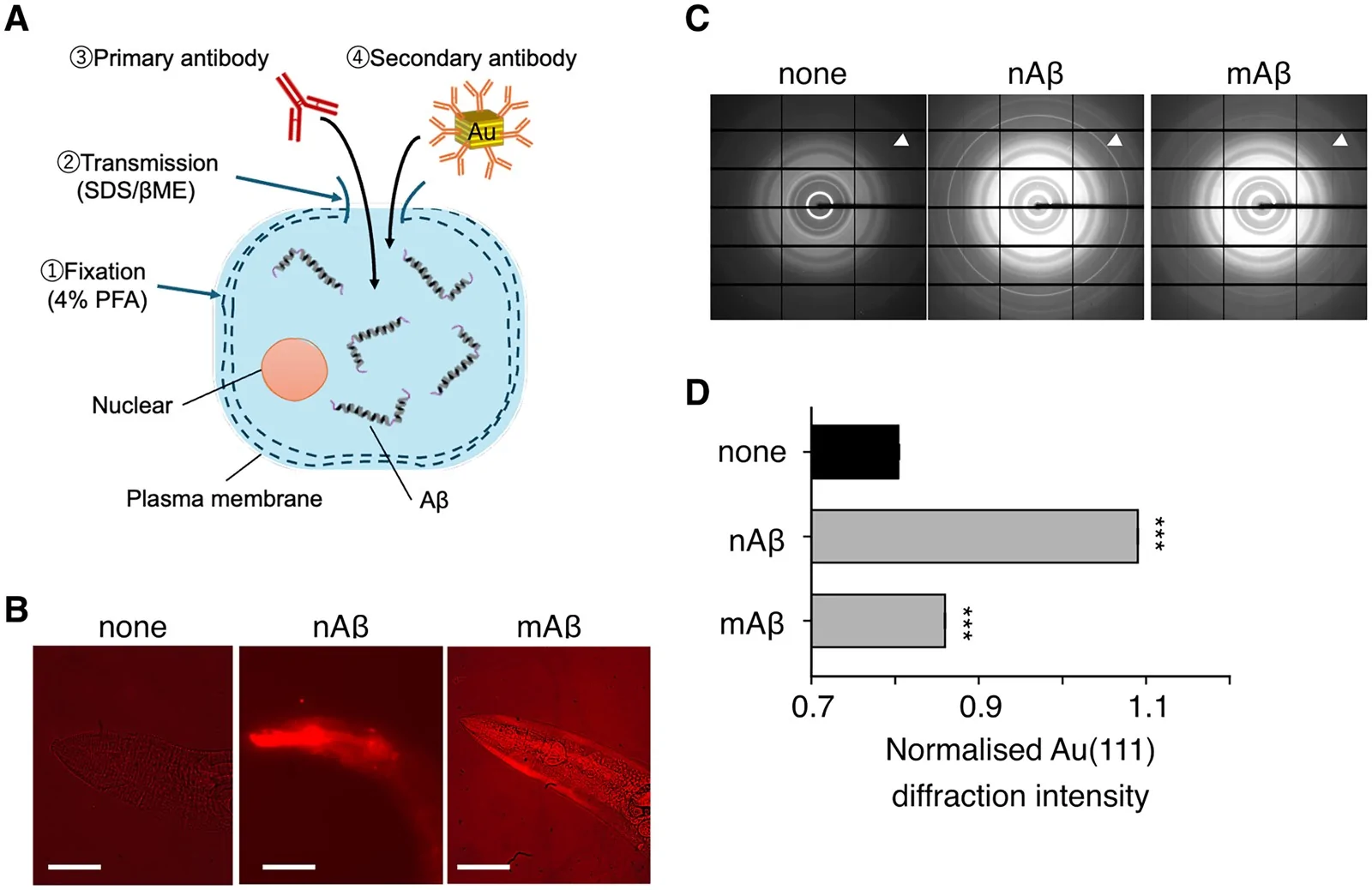
Preparation and validation of intracellular Aβ samples for DXB measurement in *C. elegans*. (A) Schematic overview of the intracellular labelling procedure for Aβ. Worms were fixed with PFA, permeabilised with SDS/βME. They were then labelled with an anti-Aβ primary antibody, followed by a gold nanoparticle-conjugated secondary antibody and approximately 60-nm gold nanoparticles for DXB measurements. (B) Fluorescence images of none (control), nAβ and mAβ worms. Scale bars, 0.1 mm. (C) Diffraction images. (D) Normalized diffraction intensity in none, nAβ, and mAβ. Data are presented as the mean ± SEM (*n* = 5). Statistical significance was assessed using Student’s *t*-test. ***P < .001.

As mentioned above, both nAβ and mAβ worms exhibited physiological abnormalities. We therefore considered these worms to provide an appropriate in-vivo sample background for evaluating Aβ-associated nanoscale dynamics. To measure intracellular molecular nanoscale dynamics in animals using DXB, we attempted to establish a method for labelling Aβ molecules in *C. elegans* with gold nanoparticles. In this study, we adapted the concept of immunofluorescence staining and examined whether intracellular Aβ could be labelled by introducing antibodies and gold nanoparticles into worm tissues after fixation and permeabilization. These Aβ worms were subjected to fixation, permeabilization, anti-Aβ antibody labelling, and gold nanoparticle labelling according to the procedure shown in Fig. 3A. To validate the labelling procedure, we first performed immunofluorescence staining using Aβ-expressing and non-Aβ-expressing worms. No clear fluorescent signal was observed in non-Aβ-expressing worms. In contrast, nAβ worms showed fluorescent signals around the head neuronal region, whereas mAβ worms showed fluorescent signals along the body-wall muscles (Fig. 3B and Fig. S1). These results confirmed the presence of Aβ in the respective transgenic worms and demonstrated that anti-Aβ antibody-based immunostaining was applicable to this sample system. Furthermore, these findings support the feasibility of using a similar antibody-mediated strategy for the gold nanoparticle labelling of Aβ for DXB measurements.

After fixation, permeabilization and anti-Aβ primary antibody labelling, the Aβ-expressing and non-Aβ-expressing control worms were subjected to gold nanoparticle labelling. Because the diffraction signal obtained with the gold nanoparticle-conjugated secondary antibody alone was weak, an additional incubation with approximately 60-nm gold nanoparticles in PBS was performed for 1 h to enhance the contrast of the Au(111) diffraction. After incubation, the worms were washed with PBS, placed on polyimide film and mounted on a sample holder. Time-resolved X-ray measurements were then performed by targeting individual worm samples, and the X-ray diffraction intensities derived from the gold nanoparticles were compared. Although non-Aβ control worms presented detectable Au(111) diffraction signals, compared with control worms, both nAβ and mAβ worms clearly exhibited stronger diffraction intensities (Fig. 3C and D). In addition, bright-field microscopy images of gold nanoparticle-treated non-Aβ-expressing and Aβ-expressing worms showed a tendency towards increased darkening in regions corresponding to the Aβ expression sites (Fig. S2). This finding further supports the possibility that gold nanoparticles preferentially accumulated in Aβ-expressing regions. These results indicate that nonspecific gold nanoparticle retention was present to some extent but that Aβ-expressing worms retained higher levels of gold nanoparticles through the antibody-mediated labelling procedure. This method therefore provides a useful sample preparation strategy for detecting Aβ-associated Au(111) diffraction signals in fixed whole-mount worms, enabling subsequent DXB measurements.

### Detection of intracellular Aβ-associated nanoscale dynamics by DXB

**Figure 4:**
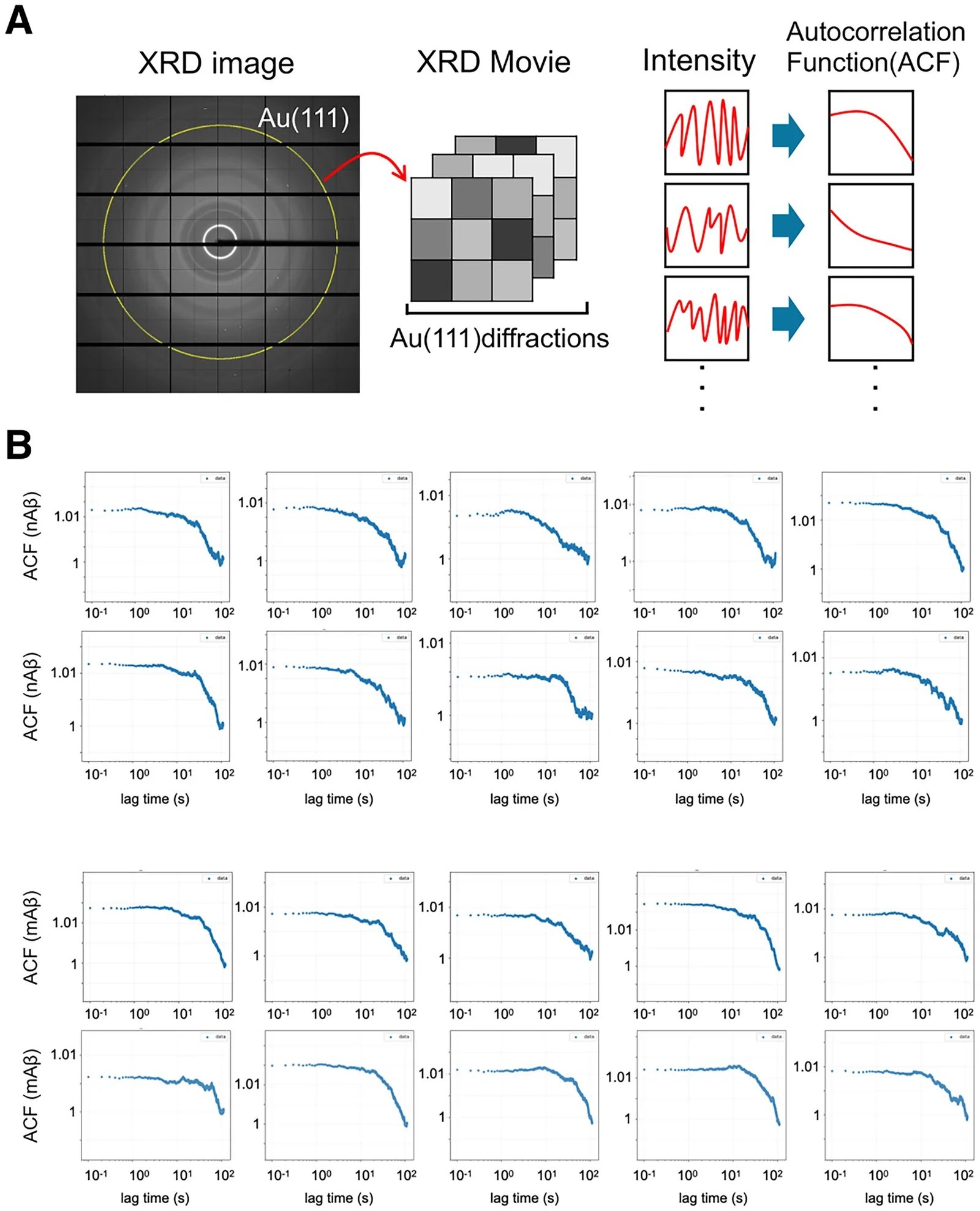
Extraction of molecular motion information from Au(111) diffraction intensity fluctuations. (A) Representative workflow of DXB analysis. Au(111) diffraction spots were extracted from time-resolved X-ray diffraction videos, and the intensity fluctuation of each diffraction spot was converted into an autocorrelation function (ACF). Differences in ACF decay profiles reflect the rotational and/or fluctuating motion of intracellular Aβ-associated regions. (B) Representative examples of diverse ACF decay curves obtained from intracellular Aβ measurements. Both the mAβ and nAβ samples presented heterogeneous ACF profiles.

To evaluate the intracellular Aβ-associated nanoscale dynamics, DXB measurements were performed using Aβ-expressing worms. Time-series diffraction intensity data were extracted from the acquired diffraction images after 10 × 10-pixel spatial binning and used for further analysis. The analysis workflow is shown in Fig. 4A. First, temporal changes in the diffraction intensity derived from Au(111) of the gold nanoparticles were extracted from each spatial bin. An ACF analysis was then applied to the obtained intensity time traces to evaluate the temporal correlation of diffraction intensity fluctuations at each spatial bin. Although all the resulting ACF curves showed an overall decay, their profiles were diverse. In addition to curves exhibiting simple exponential decay, some showed oscillatory fluctuations superimposed on the decay, whereas others exhibited more complex decay behaviour that could not be adequately described by a single-exponential function (Fig. 4B).

### PCA–LDA analysis of ACF profiles from neuronal and muscle Aβ samples

**Figure 5:**
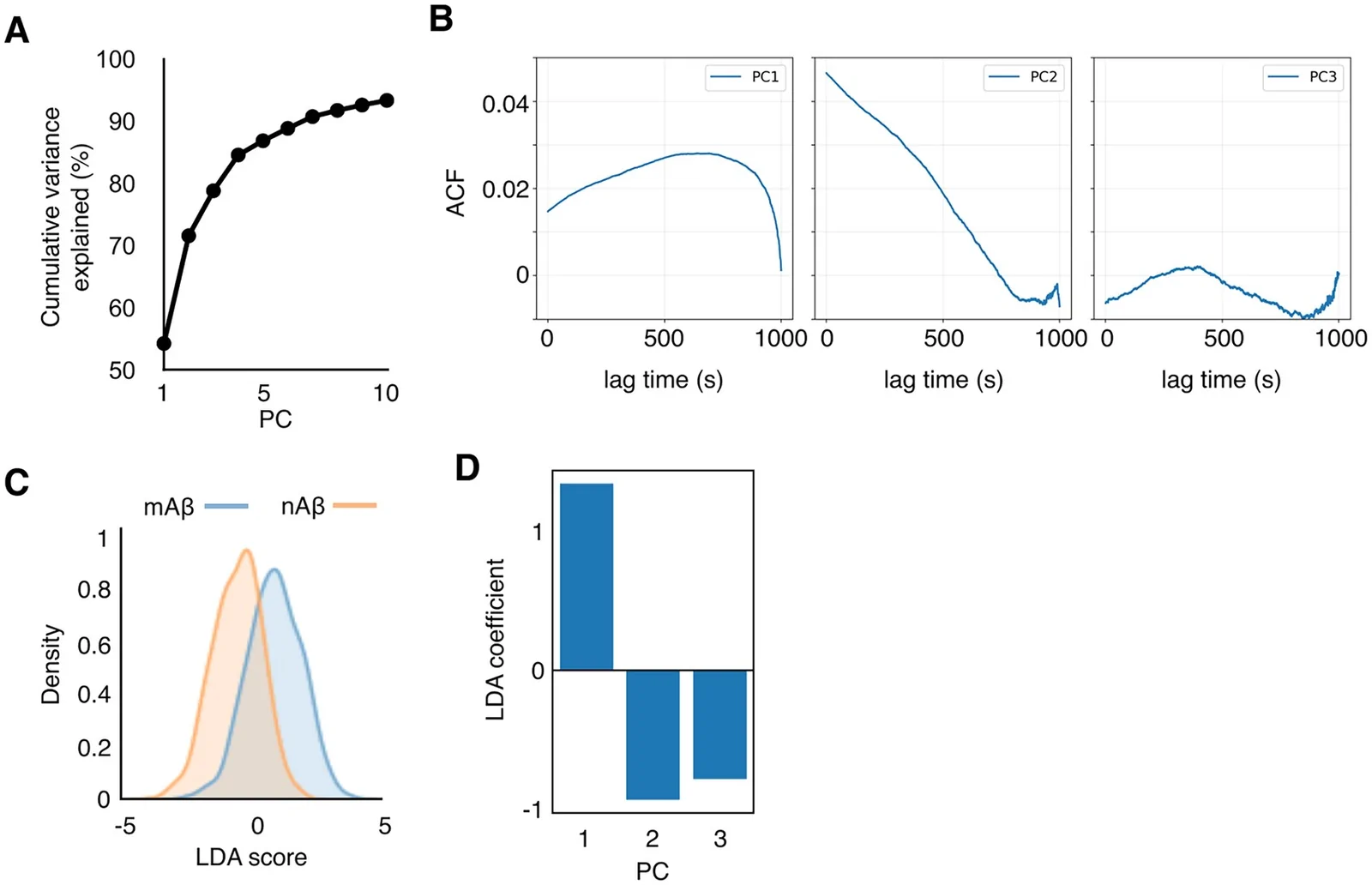
PCA–LDA analysis of ACF profiles obtained from intracellular Aβ DXB measurements. (A) Cumulative variance explained by principal components (PCs) calculated from the ACF curve data. (B) Representative loading profiles of PC1–PC3, showing characteristic temporal features within the ACF curves. (C) LDA classification of PC scores derived from the ACF profiles of mAβ and nAβ worms. (D) LDA coefficients for each PC, indicating the principal components contributing to discrimination between the sample groups.

To extract the dominant dynamic components represented in the ACF datasets, principal component analysis (PCA) was performed on all the ACF data to reduce dimensionality. The first 10 principal components explained 93.5% of the total variance in the ACF dataset (Fig. 5A). The loading profiles for PC1–PC3 are shown in Fig. 5B, while those for PC1–PC20 are provided in Fig. S3. PC1 exhibited a broad, slowly varying profile rather than a simple relaxation-like decay. PC2 showed the clearest monotonic decay-like trend. PC3 exhibited a weaker, non-monotonic profile, with a broad maximum followed by a gradual decrease and a subsequent upturn towards the end of the analysed range. These loading profiles suggest that the overall ACF dataset contained multiple components of temporal variation, including fluctuations that cannot be fully explained by a simple monotonic relaxation process.

We next examined whether the dynamic features of nAβ and mAβ could be distinguished by machine learning using PCA scores as features. LDA was performed using the PCA scores of the major principal components. The first three principal components were selected as a compact representation of the major features in the ACF profiles and were used for LDA classification. The classification accuracy was 82.8% for the training dataset and 81.5% for the test dataset (Fig. 5C). Analysis of the principal components contributing to the discrimination revealed that PC1 and PC2 made a major contribution, suggesting that the main information distinguishing nAβ and mAβ was primarily contained in the decay component of the ACF curves. These results indicate that the combination of worm DXB measurements and PCA–LDA can be used to extract nanodynamic features associated with Aβ molecules or regions near Aβ aggregates in whole-mount animal samples. These findings suggest that tissue-dependent dynamic differences may be reflected in the ACF profiles of Aβ-expressing worms.

### Quantitative comparison of the ACF decay constants and in-vitro Aβ DXB reference measurements

**Figure 6:**
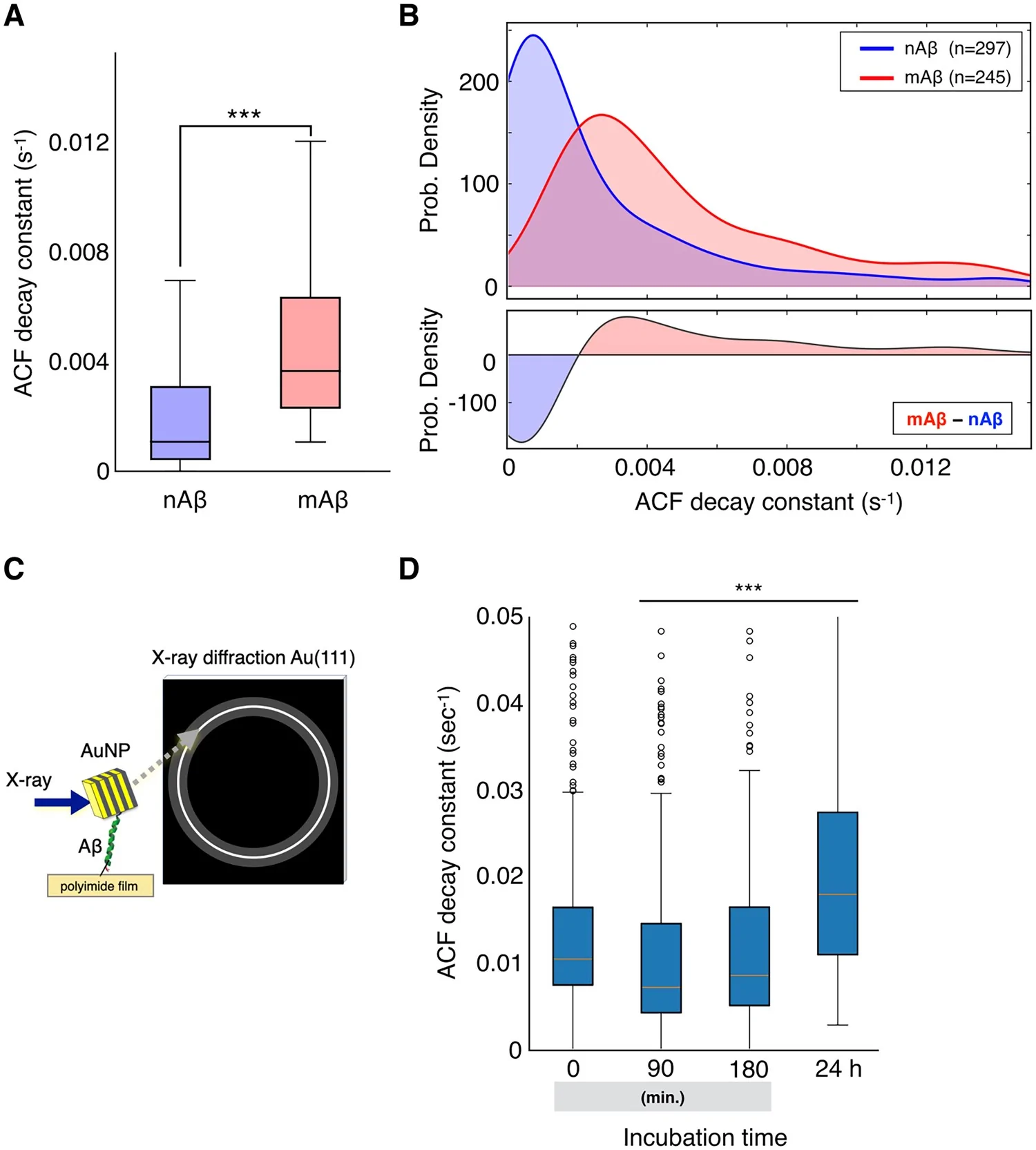
Comparison of Aβ-associated dynamics in intracellular and in vitro DXB measurements. (A) Distribution of ACF decay constants obtained from intracellular Aβ DXB measurements in neuronal (nAβ) and muscle cells (mAβ). (B) Probability density distributions of the ACF decay constants for nAβ and mAβ worms. Positive values indicate decay-constant components relatively enriched in mAβ, whereas negative values indicate components relatively enriched in nAβ. (C) Schematic illustration of in-vitro Aβ DXB. (D) Time-dependent change in the ACF decay constant of in-vitro Aβ samples. Statistical significance was assessed using the Wilcoxon rank-sum test. In the DXB analysis, n indicates the number of 10 × 10-pixel spatial bins for which the ACF curves were successfully fitted. n.s., not significant; *P < .05, ***P < .001. For the in vitro measurements, *n* = 234, 235, 233, and 156 retained spatial bins for 0 min, 90 min, 180 min, and 24 h, respectively.

PCA–LDA suggested that nAβ and mAβ differ in their ACF relaxation components. To quantitatively compare these relaxation properties, we analysed the decay constants obtained from the ACF analysis of the Au(111) diffraction intensity. Representative XRD images and the corresponding ACF decay constant maps revealed that although no marked differences between conditions were observed in the static diffraction images, ACF-derived dynamic signals were detected in control (none), nAβ, and mAβ worms (Fig. S4). These results suggest that ACF analysis can extract information on molecular dynamics that is difficult to capture using conventional XRD intensity patterns alone. As shown in Fig. 6A, box plot analysis of the decay constant distributions revealed that compared with nAβ, mAβ exhibited a broader distribution. In addition, probability density distributions of the decay constants were calculated, and the difference between mAβ and nAβ was evaluated. nAβ contained a relatively large fraction of slow relaxation components, whereas mAβ tended to contain a relatively large fraction of fast relaxation components (Fig. 6B). These results suggest that compared with Aβ expressed in the nervous system, Aβ expressed in body-wall muscle cells may contain faster molecular motion components.

It remains unclear what molecular state of Aβ is reflected by this difference in relaxation speed. Therefore, to provide reference data for interpreting the worm DXB results, we performed DXB measurements of Aβ in vitro (Fig. 6C). The ACF decay-constant distributions varied among the incubation conditions, with the 24-h sample showing higher decay constants than the 0-min sample (Fig. 6D). As Aβ is known to aggregate over time and form fibrillar structures, the increase in molecular mobility observed by in-vitro DXB may reflect changes in structural dynamics associated with the Aβ aggregation process. On the basis of these results, the increase in fast relaxation components observed in mAβ may reflect a more dynamically heterogeneous Aβ-associated state. Thus, worm DXB analysis may enable the detection of tissue-dependent differences in the relaxation dynamics of Aβ.

## Discussion

In this study, we established a DXB-based workflow for measuring Aβ-associated nanoscale dynamics in fixed and permeabilized whole-mount *C. elegans*. A key methodological advance is that intracellular Aβ targets can be labelled by antibody-mediated gold nanoparticle tagging and analysed without dissection or sectioning. This approach enabled dynamic information associated with intracellular targets to be obtained from fixed whole-organism specimens. It also demonstrated the potential to extend DXB beyond conventional isolated or simplified sample systems, such as in vitro single-molecule measurements and measurements of targets on cell-membrane surfaces, towards a method capable of evaluating target-molecule dynamics within cells, fixed whole organisms, and biological tissues.

Physiological assays revealed that both nAβ and mAβ worms exhibited abnormalities associated with Aβ toxicity. In the present study, these assays were performed primarily to confirm that the worms used for DXB analysis represented biologically relevant in-vivo samples in which Aβ expression was accompanied by physiological dysfunction, as reported previously in Aβ-expressing *C. elegans* models [17, 18, 22]. In the thrashing and egg laying assays, mAβ worms presented more pronounced phenotypes, likely reflecting impaired muscle function, which is consistent with the findings of previous studies using body-wall muscle Aβ expression models [23–25]. In contrast, nAβ-expressing worms also showed reduced thrashing, suggesting that Aβ-associated behavioural deficits are not limited to direct muscle impairment but may also involve altered neuronal regulation of locomotion [26, 27]. In the NaCl learning assay, no significant difference was detected in nAβ worms, whereas a significant reduction was observed in mAβ worms. Salt chemotaxis learning is regulated by defined sensory and interneuron circuits, including insulin/PI3K-dependent neuronal plasticity [28–31]. Thus, neuronal Aβ expression could, in principle, affect NaCl learning. In the present study, however, the learning index of the nAβ worms showed a non-significant downward trend, suggesting that this effect was not robust under the present experimental conditions. In contrast, the learning ability of the mAβ-expressing worms was impaired while basal salt chemotaxis remained largely intact, indicating that NaCl sensing and basic chemotactic ability were largely preserved. Thus, the reduced learning response in mAβ worms is unlikely to reflect a simple defect in NaCl sensing or basal chemotaxis. Rather, it may reflect impaired expression of the conditioned change in chemotactic behaviour after NaCl-starvation conditioning, potentially influenced by motor or physiological defects associated with muscle Aβ expression. This interpretation is consistent with previous work showing that locomotor changes can correlate with salt chemotaxis learning performance without directly affecting basal NaCl chemotaxis [32].

PCA–LDA suggested that nAβ and mAβ possess distinct dynamic features. Notably, LDA achieved classification accuracies of more than 81.5% for both the training and test datasets, indicating that the dynamic information extracted from the ACF profiles was highly effective in discriminating between the two sample types. However, because the PCA–LDA analysis was based on spatial-bin-level splitting, these classification accuracies should be interpreted as reflecting the separability of ACF-pattern features rather than fully independent biological prediction performance. Importantly, PC1 and PC2 contributed most strongly to the separation and corresponded to a relaxation-like component in the ACF profiles. This observation indicates that the principal difference between nAβ and mAβ was associated mainly with the dominant decay-related component, supporting the validity of focusing on the ACF decay constant as a physically interpretable parameter in this study.

The ACF relaxation behaviour, quantified by the decay constant, has been experimentally shown to reflect the mobility of gold nanocrystals, with faster ACF decay corresponding to greater mobility [1]. Although absolute quantification of molecular or nanoparticle motion by DXB remains limited, the decay constant serves as a useful parameter for assessing relative differences in nanoscale dynamics between samples or experimental conditions. In this study, the culture conditions, developmental stage, and gold-labelling procedure were kept as consistent as possible to compare the dynamics of nAβ and mAβ. The faster relaxation component detected in mAβ cannot be directly assigned to a specific aggregate species, but may reflect an Aβ-associated state with locally enhanced mobility [1, 16, 33]. The in-vitro DXB measurements provided useful reference information for interpreting the worm data. In vitro, the relaxation dynamics of Aβ increased with increasing incubation time, suggesting that local dynamic behaviour changes as Aβ assemblies evolve. These observations may support the interpretation that the dynamic differences detected in worms reflect biologically relevant changes in Aβ-associated states. However, the precise molecular basis of these differences remains unclear and requires further investigation.

The DXB signals observed in *C. elegans* may reflect not only the dynamics of Aβ itself but also tissue-dependent differences in the intracellular environment surrounding Aβ, such as molecular crowding, local viscosity, interacting partners, or assembly state [8, 10, 34]. Secondary alterations in the cytoskeleton and other cellular structures caused by Aβ toxicity may also contribute to the observed signals. Therefore, the obtained decay constants should be interpreted not as direct measures of Aβ molecular motion, but as indicators of local dynamics within Aβ-associated regions. DXB is well suited to evaluating subtle rotational and fluctuational motions on the subnanometre scale. By contrast, dynamics occurring on larger spatial scales and arising from the cytoskeleton or other cellular structures may be assessed using small-angle X-ray blinking (SAX) [35], which enables label-free dynamic measurements in the SAXS region, or transmitted X-ray blinking [21], which evaluates dynamics from X-ray transmission images. Future complementary analyses combining these approaches with DXB may enable the respective contributions of molecular-level and cellular-structure-level dynamics to be examined in greater detail.

Several limitations of the present worm DXB measurements should also be noted. First, the measurements were performed using fixed and permeabilized worms and therefore do not represent molecular dynamics in living animals under normal physiological conditions. Second, although DXB can provide information on Aβ-associated dynamics, it does not directly visualize the structural state of Aβ aggregates. Nevertheless, it may enable the assessment of local dynamics associated with structurally distinct Aβ aggregate states and their spatial distribution around Aβ fibrils. In this article, measurements were performed using X-ray beam diameters of approximately 150–800 µm. Future DXB measurements using a synchrotron X-ray microbeam may provide greater spatial resolution, allowing fluctuations in individual local regions, including regions containing Aβ fibrils, to be distinguished and evaluated separately. Third, the gold nanoparticle labelling method has several limitations. One concern is that the labelling procedure itself may alter pre-existing Aβ assemblies or induce additional aggregation. To assess this possibility, we performed in-vitro SAXS measurements to examine whether the addition of gold nanoparticles and the BC05 antibody caused large-scale changes in Aβ aggregation or association. Although differences in absolute scattering intensity were observed among the samples, the normalized scattering profiles largely overlapped, no SAXS-detectable alteration attributable to the gold nanoparticles or BC05 was observed in the Au-dominated scattering profiles (Fig. S5). However, the similarity of the SAXS profiles alone does not directly demonstrate that the β-sheet secondary structure of Aβ was preserved. Further validation using FTIR spectroscopy, circular dichroism spectroscopy, or wide-angle X-ray scattering or diffraction would therefore be valuable.

Another limitation concerns the specificity of gold labelling. Antibody-mediated gold labelling may preferentially detect accessible Aβ epitopes. Furthermore, detectable Au(111) diffraction signals were also obtained from the none group, which comprised a strain that did not express Aβ, suggesting that some nonspecific binding or retention of the gold nanoparticles occurred. In the DXB analysis, the distribution of ACF decay constants in the none group was clearly shifted towards higher values than those in the nAβ and mAβ groups (Fig. S6). This finding may indicate that the nonspecifically retained gold nanoparticles were in a relatively unconstrained and mobile state. The difference between the decay-constant distribution of the nonspecifically retained gold nanoparticles and those observed in the nAβ and mAβ groups supports the interpretation that the DXB signals obtained from the Aβ-expressing groups contained information on Aβ-associated dynamics. In addition, fluorescence X-ray mapping of the gold nanoparticle distribution, combined with its spatial overlay with fluorescence images of Aβ, would allow Aβ-specific gold nanoparticle binding to be verified more directly and robustly.

To clarify the relationships among DXB-derived dynamics, the Aβ aggregation state, and physiological dysfunction, future studies combining DXB with aggregation-specific probes, fluorescence lifetime imaging, electron microscopy, atomic force microscopy, or time-course analyses will be needed [36–38]. In particular, combining DXB with high-spatial-resolution synchrotron X-ray microdiffraction is expected to enable more informative analyses. Several studies have reported the structural characterization of amyloid aggregates, including Aβ fibrils and tau aggregates, using microdiffraction based on the characteristic structural signatures of β-sheets [39–41]. By contrast, DXB does not directly identify molecular structures but is characterized by its ability to assess Aβ-associated dynamics. The two techniques therefore provide distinct and complementary information. Integrating structural information obtained by microdiffraction with dynamic information obtained by DXB may clarify the relationship between structural and dynamic changes during Aβ association, aggregation, and fibril formation, as well as how these changes ultimately contribute to the formation of distinct aggregate states.

## Supplementary Material

bpag044_Supplementary_Data

## Data Availability

The data underlying this article will be shared on reasonable request to the corresponding author.
