# Supplementary material for: Whole-mount diffracted X-ray blinking analysis of intracellular amyloid-β-associated nanoscale dynamics in Caenorhabditis elegans
Source: Biol Methods Protoc. 2026 Aug 13;11(1):bpag044. doi: 10.1093/biomethods/bpag044 (PMC13570790; doi:10.1093/biomethods/bpag044)
Supplement: Supplementary file 1 [file bpag044_supplementary_data.pdf]

Supplemental Information:

Whole-Mount Diffracted X-ray Blinking Analysis of Intracellular Amyloid-β-Associated Nanoscale Dynamics in *Caenorhabditis elegans*

Kotaro Ozaki[1], Yoichi Shinkai[2], Yuji C. Sasaki[3], Masahiro Kuramochi[1,3,*]

[1]Graduate School of Science and Engineering, Ibaraki University, Hitachi, 316-8511, Japan

[2]Cellular and Molecular Biotechnology Research Institute, National Institute of Advanced Industrial Science and Technology (AIST), Tsukuba, 305-8566, Japan

[3]Graduate School of Frontier Sciences, The University of Tokyo, Kashiwa, 277-8561, Japan

[*]Correspondence email: masahiro.kuramochi.vw26@vc.ibaraki.ac.jp

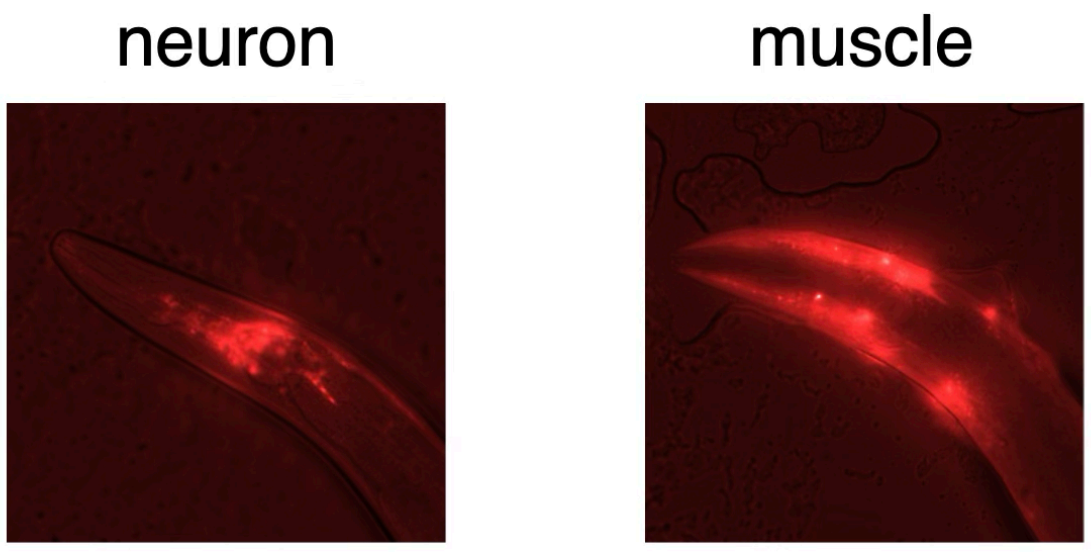

Fig. S1. Fluorescence images of neuronal and body-wall muscle expression patterns in *C. elegans*. Representative fluorescence images of transgenic *C. elegans* showing fluorescence in neurons (CMS50) and body-wall muscle (CMS95). These strains do not express Aβ and were used to visualise fluorescence patterns comparable to those of the Aβ-expressing strains shown in Fig. 3B.

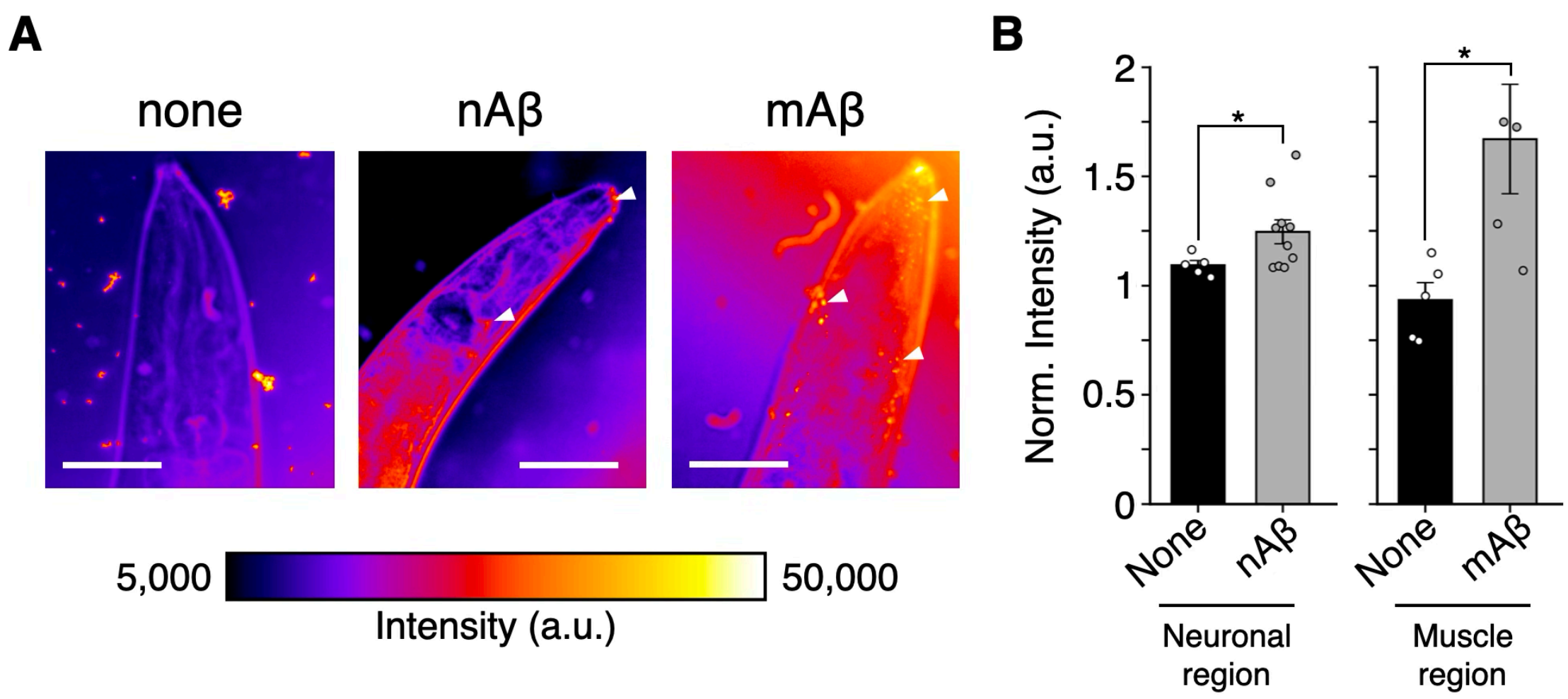

Fig. S2. Analysis of gold nanoparticle distribution in Aβ-expressing and control (none) worms using bright-field microscopy. (A) Representative bright-field microscopy images of the non-Aβ-expressing control worm (none), the neuronal Aβ-expressing worm (nAβ), and the muscle Aβ-expressing worm (mAβ) after gold nanoparticle treatment. Because regions containing gold nanoparticles exhibited lower image intensities, the images were intensity-inverted for quantitative comparison and displayed in pseudocolour. Putative gold nanoparticle-containing regions are indicated by white arrowheads. Scale bars, 0.1 mm. (B) Mean pixel intensities were quantified within defined neuronal and muscle regions of interest (ROIs). The neuronal ROI was defined at the nose tip, whereas the muscle ROI was defined along the peripheral body-wall muscle region. Following intensity inversion, the values were normalised by division by the background intensity measured in adjacent regions within the worms. Data are presented as the mean ± standard error (SE) (n = 5–10). Statistical significance was assessed using Student's t-test. $*p < 0.05$.

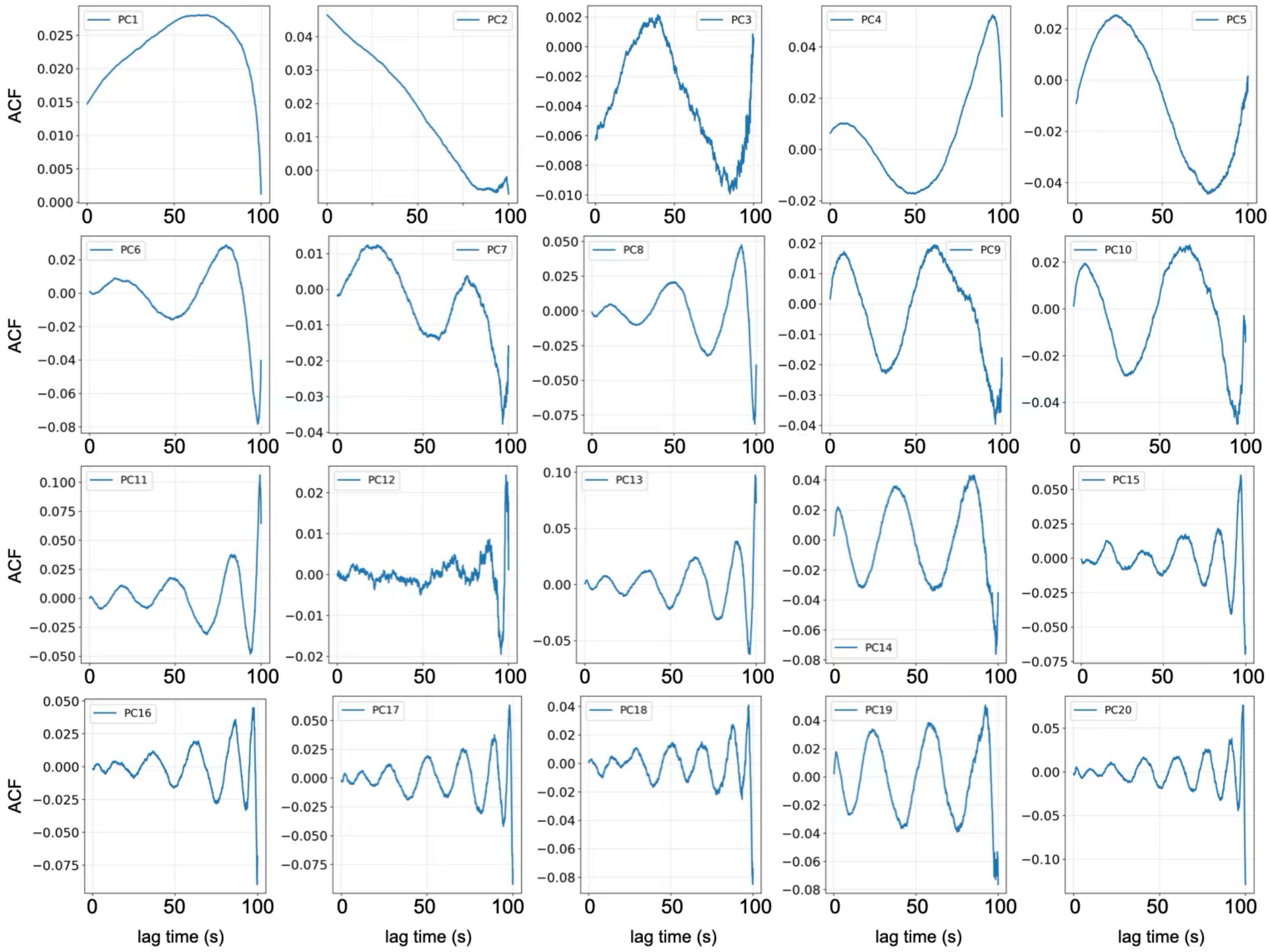

Fig. S3. Principal-component loading profiles derived from the ACF data. Loading profiles of the first 20 principal components are shown as a function of lag time. Although only the first three principal-component scores were used for the subsequent PCA–LDA classification, a larger number of components are presented here to provide a broader overview of the temporal features captured by the PCA.

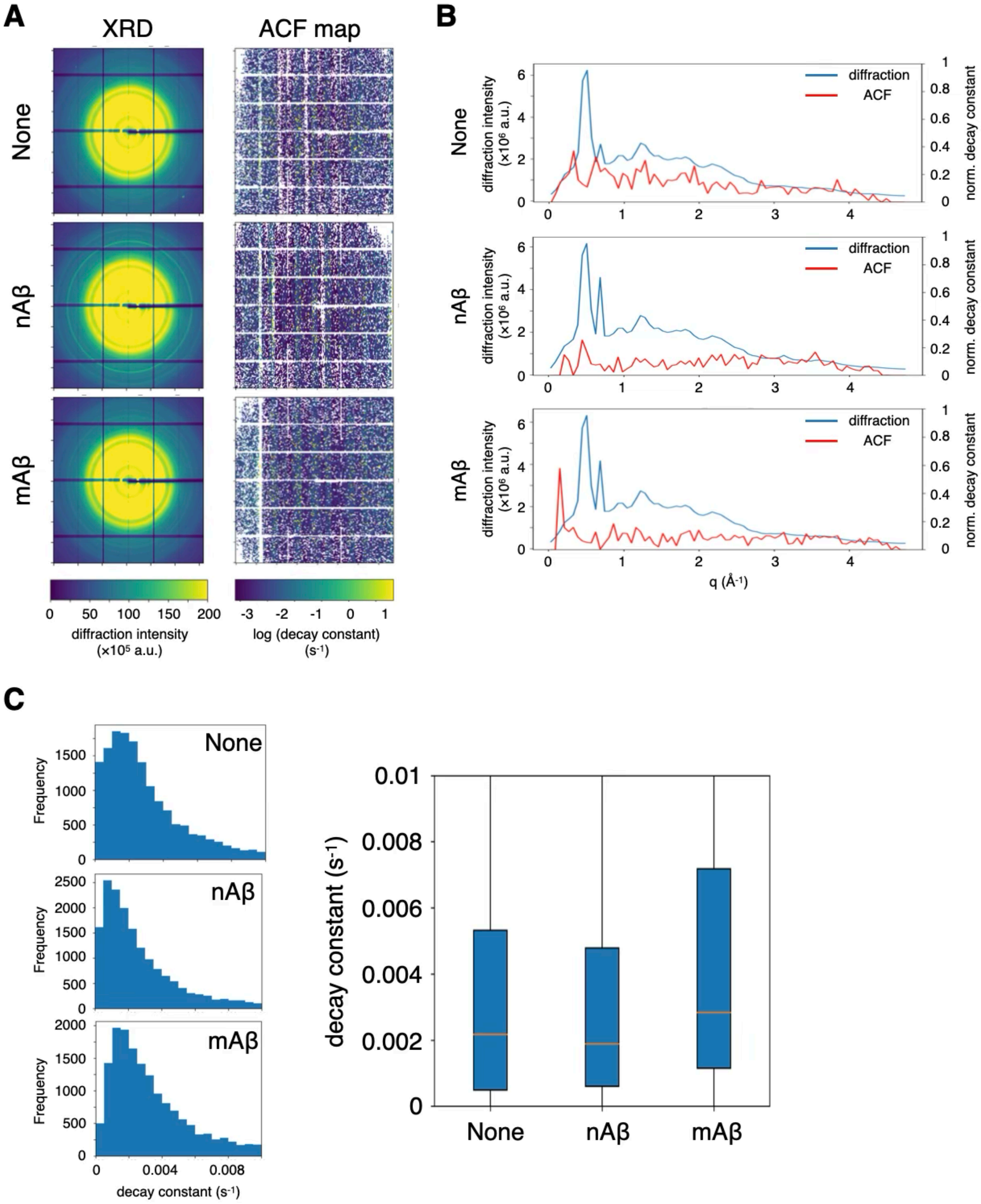

Fig. S4. X-ray diffraction images and ACF-derived dynamic maps of Aβ-expressing *C. elegans*. (A) Representative XRD images and corresponding ACF maps from control (none), nAβ, and mAβ worms. The ACF maps show the spatial distribution of decay constants calculated from time-resolved diffraction intensity

fluctuations. (B) Radial profiles of diffraction intensity and ACF decay constants as a function of the scattering vector q. (C) Histograms and box plots of ACF decay constants obtained from control, nAβ, and mAβ worms.

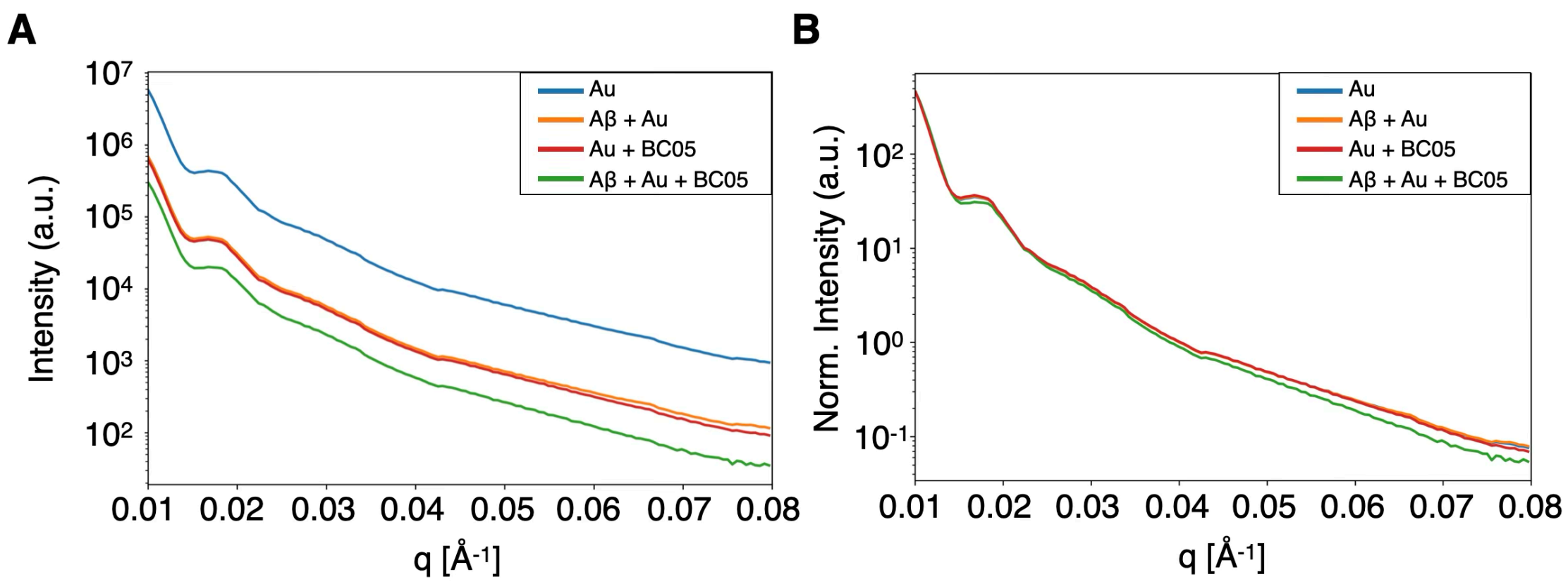

Fig. S5. SAXS profiles of gold (Au) nanoparticles, Aβ, and BC05 mixtures. (A) Background-subtracted SAXS profiles of Au, Aβ + Au, Au + BC05, and Aβ + Au + BC05. (B) Normalised SAXS profiles used to compare the shapes of the scattering curves. Although the absolute scattering intensities differed among the samples, the normalised profiles largely overlapped.

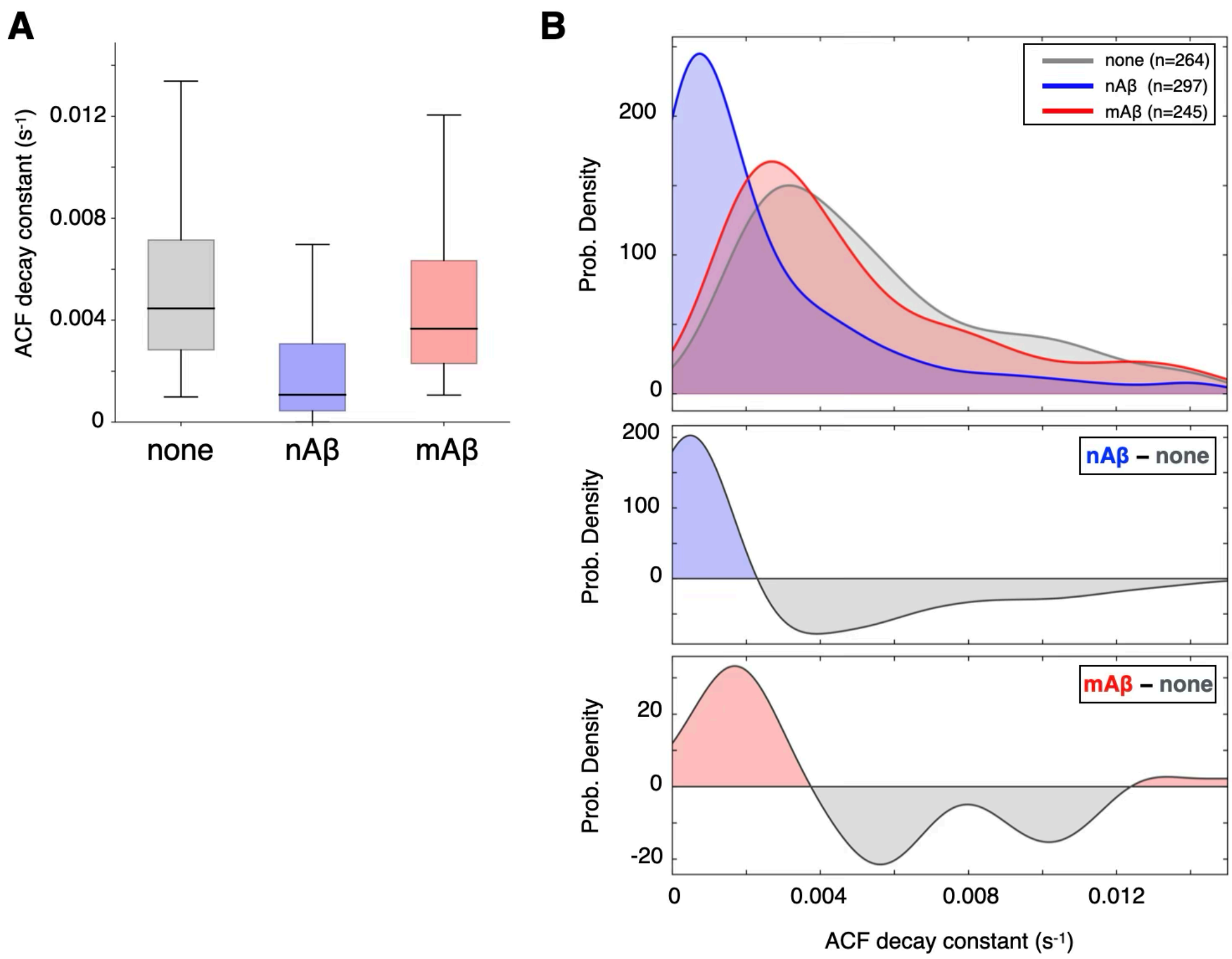

Fig. S6. Comparison of ACF decay-constant distributions for nonspecific and Aβ-associated gold nanoparticles. (A) Boxplots of the ACF decay constants obtained from the none, nAβ, and mAβ groups. (B) Probability density distributions of the ACF decay constants for none, nAβ, and mAβ (upper panel), and differences in probability density between nAβ and none and between mAβ and none (lower panels). Positive values indicate components that were relatively more abundant in the Aβ-expressing groups, whereas negative values indicate components that were relatively more abundant in none.
